# Biological Assessment of a ^18^F-Labeled Sulforhodamine 101 in a Mouse Model of Alzheimer’s Disease as a Potential Astrocytosis Marker

**DOI:** 10.3389/fnins.2019.00734

**Published:** 2019-07-16

**Authors:** Ingrid Kreimerman, Ana Laura Reyes, Andrea Paolino, Tania Pardo, Williams Porcal, Manuel Ibarra, Patricia Oliver, Eduardo Savio, Henry Engler

**Affiliations:** ^1^Radiopharmacy Department, Uruguayan Centre of Molecular Imaging (CUDIM), Montevideo, Uruguay; ^2^Department of Organic Chemistry, Faculty of Chemistry, University of the Republic (UdelaR), Montevideo, Uruguay; ^3^Pharmaceutical Sciences Department, Faculty of Chemistry, University of the Republic (UdelaR), Montevideo, Uruguay

**Keywords:** [^18^F]2B-SRF101, [^11^C]deuterodeprenyl, PET radiopharmaceutical, astrocytosis, Sulforhodamine 101, astrocyte tracer

## Abstract

Neurodegenerative diseases have mainly been associated with neuronal death. Recent investigations have shown that astroglia may modulate neuroinflammation in the early and late stages of the disease. [^11^C]Deuterodeprenyl ([^11^C]DED) is a tracer that has been used for reactive astrocyte detection in Alzheimer’s disease, Creutzfeldt–Jakob disease and amyotrophic lateral sclerosis, among others, with some limitations. To develop a new radiotracer for detecting astrocytosis and overcoming associated difficulties, we recently reported the synthesis of a sulfonamide derivative of Sulforhodamine 101 (SR101), labeled with ^18^F, namely SR101 *N*-(3-[^18^F]Fluoropropyl) sulfonamide ([^18^F]2B-SRF101). The red fluorescent dye SR101 has been used as a specific marker of astroglia in the neocortex of rodents using *in vivo* models. In the present work we performed a biological characterisation of the new tracer including biodistribution and micro-PET/computed tomography (CT) images. PET/CT studies with [^11^C]DED were also done to compare with [^18^F]2B-SRF101 in order to assess its potential as an astrocyte marker. Biodistribution studies with [^18^F]2B-SRF101 were carried out in C57BL6J black and transgenic (3xTg) mice. A hepatointestinal metabolization as well as the pharmacokinetic profile were determined, showing appropriate characteristics to become a PET diagnostic agent. Dynamic PET/CT studies were carried out with [^18^F]2B-SRF101 and [^11^C]DED to evaluate the distribution of both tracers in the brain. A significant difference in [^18^F]2B-SRF101 uptake was especially observed in the cortex and hippocampus, and it was higher in 3xTg mice than it was in the control group. These results suggested that [^18^F]2B-SRF101 is a promising candidate for more extensive evaluation as an astrocyte tracer. The difference observed for [^18^F]2B-SRF101 was not found in the case of [^11^C]DED. The comparative studies between [^18^F]2B-SRF101 and [^11^C]DED suggest that both tracers have different roles as astrocytosis markers in this animal model, and could provide different and complementary information at the same time. In this way, by means of a multitracer approach, useful information could be obtained for the staging of the disease.

## Introduction

Alzheimer’s disease (AD) is a progressive neurodegenerative disorder. It is the most common form of dementia in the aging population, and it has no prevention or cure (James et al., 2015). The disease is clinically characterized by memory loss, dementia, and progressive cognitive impairment. Anatomopathological changes include the presence of amyloid plaques containing amyloid-β peptide fibrils and hyperphosphorylated tau neurofibrillary tangles. The physiopathological changes are characterized by neurotransmitter deficits (Mukaetova-Ladinska et al., 1993; Näslund et al., 2000; Nordberg, 2004; Archer et al., 2006). The widespread neuronal degeneration is accompanied by widespread astrocytosis, surrounding amyloid plaques (Overmyer et al., 1999; Kadir et al., 2011). New imaging technologies are important for detecting the disease in the early stages, following the evolution of the disease, and evaluating possible treatments.

Molecular imaging by positron emission tomography (PET) is a minimally invasive technique that generates three-dimensional (3D) images of physiological processes at the molecular and cellular levels (Li and Conti, 2010). 2-Fluoro-2-deoxy-D-glucose ([^18^F]FDG) is one of the most frequently employed radiotracers for different pathologies’ diagnosis. It is useful in the estimation of brain regional glucose metabolism, as it is an analog of glucose normally consumed by the brain. In typical cases of AD, declines of glucose metabolism (hypometabolism) and cerebral blood flow initially occur in the posterior parietotemporal cortex and posterior cingulate cortex, which is associated with various vulnerability factors. Although [^18^F]FDG PET is a useful diagnostic tool for AD, its diagnostic value is limited, as regional hypometabolism is not a specific finding of AD. Thus, several PET tracers for targeting specific aspects of AD have been developed (Shimojo et al., 2015; Villemagne et al., 2018). The first was [^18^F]FDDNP, a PET tracer developed by Barrio et al. (1999) that binds non-selectively to both Aβ plaques and neurofibrillary tangles in AD. The second was the ^11^C-labeled Pittsburgh compound B ([^11^C]PIB), which binds to β-sheets in Aβ plaques with high affinity (Engler et al., 2004; Klunk et al., 2004). More recently, other ^18^F-labeled amyloid PET tracers have been developed, which specifically bind to amyloid aggregates and not neurofibrillary tangles (Fodero-Tavoletti et al., 2012; Teipel et al., 2015). Several tau tracers have also been developed and described in preclinical and clinical studies (Villemagne et al., 2015). These tracers are currently in clinical trials, and the US Food and Drug Administration (FDA) has not yet approved any of them.

For a long time, neurodegenerative diseases have mainly been associated with neuronal death. Recent investigations have shown that astroglia may modulate neuroinflammation in the early and late stages of the disease (Verkhratsky et al., 2010). Monoamine oxidase B (MAO-B), an enzyme overexpressed in reactive astrocytes, is blocked irreversibly by [^11^C]deuterodeprenyl ([^11^C]DED). This tracer has been used for detection of reactive astrocytes in AD (Santillo et al., 2011; Carter et al., 2012; Choo et al., 2014), Creutzfeldt–Jakob disease (Engler et al., 2003, 2012) and amyotrophic lateral sclerosis (Johansson et al., 2007), among others.

One of the limitations of this radiotracer is that levels of MAO-B decrease up to 40% in smokers relative to non-smokers or former smokers. This reduction of MAO-B activity influences the pattern of brain biodistribution, thereby complicating the image interpretation (Fowler et al., 1996). Quantification of [^11^C]DED binding to MAO-B, as well as the initial tracer distribution, has been analyzed via the Patlak method (Patlak et al., 1983), which has been previously described (Engler et al., 2003) indicating that the intercepts provide a measure of the blood flow, and the slope is a measure of the MAO-B binding. Despite the value of this double information obtained with only one tracer, the imaging analysis results are more complex.

To develop a new agent for detecting astrocytosis and overcoming the difficulties previously described, we recently reported the synthesis of a sulfonamide derivative of Sulforhodamine 101 (SR101) labeled with ^18^F, namely SR101 *N*-(3-[^18^F]Fluoropropyl) sulfonamide ([^18^F]2B-SRF101). *In vitro* studies of this compound indicated physicochemical and biological properties appropriate for neuroimaging (Kreimerman et al., 2017). The red fluorescent dye, SR101, has been used as a specific marker of astroglia in the neocortex of rodents using *in vivo* models (Nimmerjahn et al., 2004; Nimmerjahn and Helmchen, 2012). SR101, Sulforhodamine B and G have also been reported for *in vivo* staining of astrocytes after intravenous (i.v.) injection in rodents (Vérant et al., 2008; Appaix et al., 2012). However, the dye uptake processes are still under discussion, being recently identified the thyroid hormone transporter OATP1C1 as the SR101-uptake transporter in hippocampus and cortex (Schnell et al., 2012, 2015). Different authors have postulated that this dye labels not only astrocytes, but also mature myelinating oligodendrocytes, diffusing via gap junctions from astrocytes to oligodendrocytes. Thus, it has been proposed that astrocytes are labeled before oligodendrocytes (Wasseff and Scerer, 2011; Hill and Grutzendler, 2014; Anderova et al., 2016; Hagos and Hülsmann, 2016). We have confirmed 2B-SRF101’s ability to detect astrocytes in cell cultures with a higher affinity than that of SR101 (Kreimerman et al., 2017). In the present work we performed a biological characterisation of the new tracer including biodistribution and micro-PET/computed tomography (CT) images. PET/CT studies with [^11^C]DED were also done to compare with [^18^F]2B-SRF101 in order to assess its potential as an astrocyte marker.

## Materials and Methods

### Animals

Astrocytosis was assessed in 9- to 10-month-old mice. A male triple-transgenic mouse (3xTg) model of AD (PS1M146V, APPSwe, and tauP301L) (Oddo et al., 2003) was used for *ex vivo* and *in vivo* studies. Aged-matched C57BL6J black mice were used as controls. The animals were housed under 12:12-h light/dark cycles; food and water were given *ad libitum* in rooms with controlled temperature and humidity at the Uruguayan Centre of Molecular Imaging (CUDIM) animal facility. The research protocol was carried out in accordance with the National Bioethics Committee requirements and under the current ethical regulations of the national law on animal experimentation no. 18.611 (National Commission of Animal Experimentation, CNEA, Uruguay). CUDIM’s Animal Bioethics Committee approved these protocols.

### Radiochemical Synthesis

The synthetic processes were performed using automated synthetic platforms, namely GE TRACERlab^®^ FX-FN (^18^F-labeling) and GE TRACERlab^®^ FX C Pro (^11^C-labeling). Radionuclides were produced in a PET Trace 16.5 MeV cyclotron (GE Healthcare).

[^18^F]2B-SRF101 was synthesized according to the method previously reported by our group (Kreimerman et al., 2017). A solution of the precursor SR101 *N*-(3-Bromopropyl) sulfonamide (1 mg in 1 mL of dimethyl sulfoxide, DMSO) was added to a preactivated and dried [^18^F]-fluoride solution. The labeling was carried out at 160°C for 10 min. After labeling, the crude reaction mixture was diluted and injected into a semipreparative HPLC for purification (see Table 1). The fraction corresponding to [^18^F]2B-SRF101 was collected and diluted in water. The product was then purified using a Sep-Pak^®^ C_18_ light cartridge (Waters). Finally, formulation was carried out, and the solution was passed through a 0.2-μm hydrophilic sterilizing filter (Millex-LG, Millipore). The radiochemical purity of the end product was analyzed via analytical HPLC (see Table 1); it exceeded 95%, while the specific activity was variable (15.7–405.2 GBq/μmol) at the end of synthesis.

**TABLE 1:** Conditions employed for semipreparative and analytical HPLC.

**Compound**	**Eluent**	**Flow rate (mL/min)**	**Retention time (min)**	**Ultraviolet (UV) detection (nm)**
**Analytical**				
[^18^F]2B-SRF101	Na_2_HPO_4_ 100 mM, pH 6.8 (A) and MeCN (B); gradient: ^*^0–10 min: from 0 to100% B ^*^10–15 min: 100% B	1.0	12.8	578
[^11^C]DED	TFA 0.1%:MeCN (75:25; v/v), isocratic flow	1.5	5.4	210
**Preparative**				
[^18^F]2B-SRF101	MeCN:H_2_O (47:53; v/v)	4.0	10–13	200
[^11^C]DED	MeCN:AcONH_4_ 0.1 M (60:40; v/v)	6.0	5–6	220

^*^MeCN: Acetonitrile. The semipreparative HPLC was carried out in the synthetic platforms using a VP 250/10 mm Nucleosil 100-5 C_18_ column (Macherey–Nagel). Analytical HPLC was performed with a Shimadzu UFLC equipped with diode array and gamma detectors, using an EC 250/4.6 mm Nucleodur 100-5 C_18_ec column (Macherey–Nagel).

[^11^C]DED was synthesized according to the method previously reported by our group (Buccino et al., 2016). [^11^C]CH_3_OTf was bubbled into the organic precursor solution to react with 1 mg of the precursor nor-DED.HCl in 350 μL butanone (MEK) for 1 min at 80°C. The addition of 3 μL of 3 M NaOH was needed to neutralize the hydrochloride form of the precursor. The crude product was separated from impurities using semipreparative HPLC (see Table 1). The fraction containing [^11^C]DED was collected in water and further purified using a Sep-Pak C_18_ light cartridge (Waters). Formulation was done and the solution was passed through a 0.22-μm sterilizing filter (Millex-GV, Millipore). The radiochemical purity of the resultant compound (determined by HPLC, Table 1) exceeded 95%, and the specific activity was variable (37.0–918.1 GBq/μmol) at the end of synthesis.

### Biological Studies

*Ex vivo* fluorescence imaging was performed with the unlabeled 2B-SRF101 as a screening method to determine whether this new compound could cross the BBB (see Supplementary Material).

*Ex vivo* and *in vivo* studies were done with [^18^F]2B-SRF101 in the mouse model previously described in section “Animals.”

[^11^C]DED was used as a well-known astrocyte radiomarker to assess the performance of the new tracer in the PET studies.

#### *Ex vivo* Studies: Biodistributions

The biological profile and pharmacokinetics of [^18^F]2B-SRF101 were determined via biodistribution studies in the 3xTg mouse model and control group. Both animal models were injected i.v. with 1.5–38.2 MBq (100–250 μL) of [^18^F]2B-SRF101 into the caudal tail vein and sacrificed by cervical dislocation at 14, 30, 65, and 110 min after injection (*n* = 2–4 animals in each injection time). Organs, tissues, and blood samples were obtained (lung, heart, spleen, liver, kidneys, muscle, bone, stomach, gut, bladder, and brain), weighed and assayed for radioactivity in a gamma counter (a 3^″^ × 3^″^ well-type NaI [Tl] solid scintillation detector coupled to a multichannel analyser, ORTEC). The total urine volume was collected during the biodistribution period and removed from the bladder after sacrifice, and its activity was measured. The percent of injected dose per gram of tissue (%ID/g) and injected dose in the whole organ (%ID) was calculated. Corrections by different sample geometry were applied when necessary.

#### *In vivo* Studies: Micro-PET/CT Imaging

##### Image acquisition

Small animal PET imaging was performed in a tri-modality PET/SPECT/CT scanner (Triumph^*TM*^, TriFoil, Inc., United States), based on the Quad-APD detector modules coupled with LYSO/LGSO scintillators. This scanner has a spatial resolution of 1.0 mm and an axial field of view (FOV) of 3.75 cm.

The data were acquired in list mode in a 184 × 184 × 31 matrix with a pixel size of 0.25 × 0.25 × 1.175 mm and a coincidence window width of 22.22 ns. The animals were anaesthetized with 2% isofluorane in an oxygen flow of 2 L/min, placed in prone position on the scanner bed and injected i.v. via the caudal vein tail with 100–200 μL of [^18^F]2B-SRF101 (21.7–39.3 MBq; *n* = 7 in each animal model) or [^11^C]DED (11.5–28.0 MBq; *n* = 6 in each animal model). Dynamic brain PET studies were performed during 90 or 60 min after i.v. injection of [^18^F]2B-SRF101 (one frame × 5 min, two frames × 30 min, and one frame × 25 min) or [^11^C]DED (one frame × 5 min, one frame × 30 min, and one frame × 25 min), respectively. Sinograms were reconstructed using 3D maximum likelihood expectation maximization (MLEM) with 30 iterations.

##### Image analysis

Semiquantitative analysis was done using PMOD software, v. 3.301 (PMOD Technologies, Ltd., Zurich, Switzerland). PET studies were co-registered with the corresponding CT scan studies for anatomical localisation. The images were displayed as coronal, sagittal, and axial slices. Using the PFUS module, the brain images were spatially normalized to the mouse brain magnetic resonance imaging (MRI) template included in the PMOD software, to scale the images to the Paxinos and Watson coordinate system (Paxinos and Watson, 1998). The images were previously masked to exclude extracerebral activity and enhance the normalization data. The inverse mathematical transformation was estimated and applied to the volumes-of-interest (VOIs) included in the PMOD mouse brain atlas to fit the VOIs for each animal. An averaged 35- to 65-min frame was used for quantifying the radiotracer uptake. The average activity per volume unit (kBq/cc) was subsequently corrected for injected radioactivity and mouse weight and expressed in standardized uptake value (SUV) units. Eight VOIs were selected for further evaluation, as follows: the striatum, cortex, hippocampus, hypothalamus, thalamus, amygdala, olfactory bulb, and midbrain. The cerebellum was used as a reference, and the target to cerebellum ratio was calculated for each VOI. The results were expressed as the mean of the semi-quantitative target to cerebellum ratio in SUV ratio (SUVR) units for each VOI ± standard error of the mean (SEM).

### Statistical Analyses

In the PET/CT image analysis, data between groups were analyzed by the non-parametric Mann–Whitney *U* test, using a two-tailed test. Significance was accepted at the 95% probability level.

## Results

### *Ex vivo* Studies: Biodistributions

**FIGURE 1:**
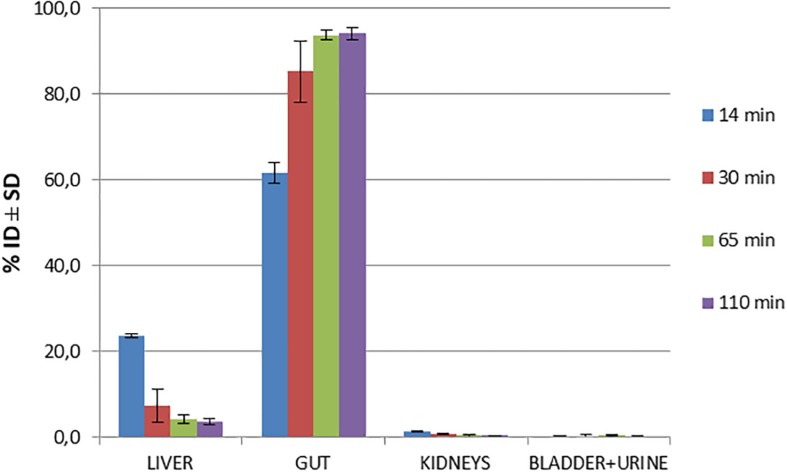
Biodistribution of [^18^F]2B-SRF101 in healthy C57BL6J black mice at different injection times.

**FIGURE 2:**
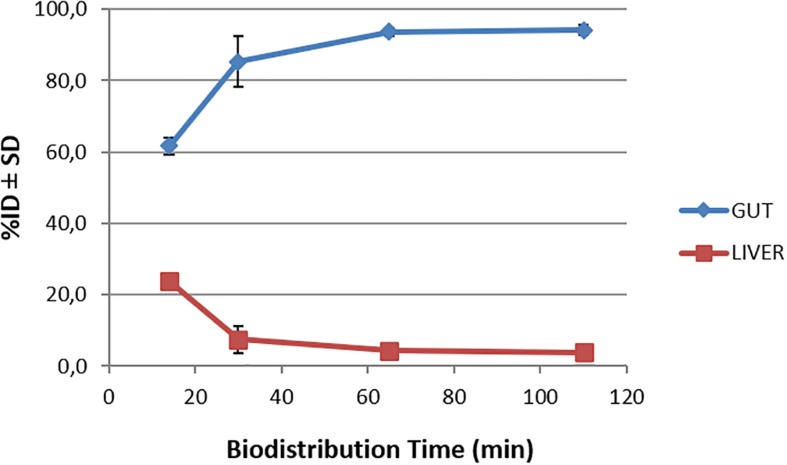
Time-activity curves for the liver and gut after injection of [^18^F]2B-SRF101 until 110 min.

Biodistribution studies with [^18^F]2B-SRF101 were carried out in C57BL6J black mice to determine its pharmacokinetic profile. The results are summarized in Figure 1 and Table 2. The tracer uptakes in the liver and gut at different injection times of [^18^F]2B-SRF101 are shown in Figure 2. [^18^F]2B-SRF101’s activity in the gut was relatively high at 14 min post-injection (p.i.; 61.7 ± 2.4%ID), reaching a plateau of 85.3 ± 7.1%ID after 30 min p.i.; this remained essentially constant over the entire 110-min study period.

**TABLE 2:** Biodistribution of radioactivity after intravenous (i.v.) injection of [^18^F]2B-SRF101 in healthy C57BL6J black mice.

**Tissue**	**14 min**	**30 min**	**65 min**	**110 min**
Blood	1.09±0.26	0.37±0.21	0.07±0.02	0.16±0.08
Liver	12.85±2.74	4.01±2.39	2.24±0.33	2.06±0.65
Heart	0.14±0.01	0.03±0.01	0.01±0.003	0.02±0.02
Lung	0.62±0.19	0.25±0.08	0.09±0.03	0.10±0.06
Spleen	0.14±0.09	0.04±0.004	0.02±0.008	0.01±0.009
Kidney	3.49±0.002	2.10±0.29	0.89±0.32	0.96±0.04
Muscle	0.27±0.27	0.03±0.02	0.01±0.002	0.01±0.004
Bone	0.07±0.06	0.07±0.004	0.03±0.008	0.03±0.009
Brain	0.11±0.04	0.07±0.01	0.03±0.003	0.04±0.01

*Results are expressed as mean %ID/g ± SD for *n* = 2 (14 and 110 min) and *n* = 3 (30 and 65 min).*

**FIGURE 3:**
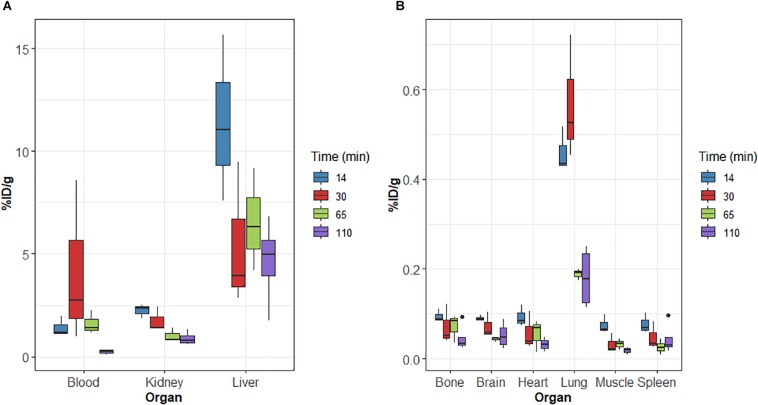
**(A,B)** Biodistribution of radioactivity after intravenous (i.v.) injection of [^18^F]2B-SRF101 in 3xTg mice. Results expressed as %ID/g for *n* = 3 (14, 30, and 65 min) and *n* = 4 (110 min).

Biodistribution studies were also performed in the 3xTg mouse model of AD at 14, 30, 65, and 110 min after [^18^F]2B-SRF101 injection. The results are summarized in Figure 3.

On relation to metabolisation, similar results were obtained for 3xTg than those of the control group. Initially, [^18^F]2B-SRF101’s activity in the gut was 61.1 ± 3.5%ID at 14 min p.i., reaching a value of 79.7 ± 16.6%ID after 110 min p.i.

Brain uptake at 65 min p.i. was compared between the 3xTg mice and control group. A higher uptake was found in the case of the 3xTg group, and the difference was significant (*p* < 0.05). Timepoint selection was performed, considering that PET/CT analysis was carried out in the frame covering from 35 to 65 min of the study.

### *In vivo* Studies: Micro-PET/CT Imaging

Dynamic PET/CT studies were carried out with [^18^F]2B-SRF101 and [^11^C]DED to evaluate the distribution of the tracers in the brain. For the analysis and quantification, the cerebellum was used as the reference because astrocytes are sparse in this region.

**FIGURE 4:**
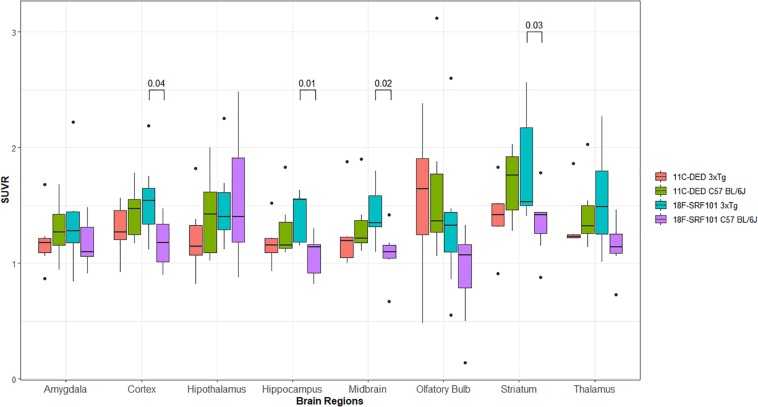
Standardized uptake value ratio (SUVR) of different brain regions analyzed between 35 and 65 min, from micro-PET/CT images with: [^18^F]2B-SRF101 in 3xTg mice (*n* = 7) and controls (*n* = 7) and [^11^C]DED in 3xTg mice (*n* = 6) and controls (*n* = 6). Significant difference between groups are indicated by *p* < 0.05.

**FIGURE 5:**
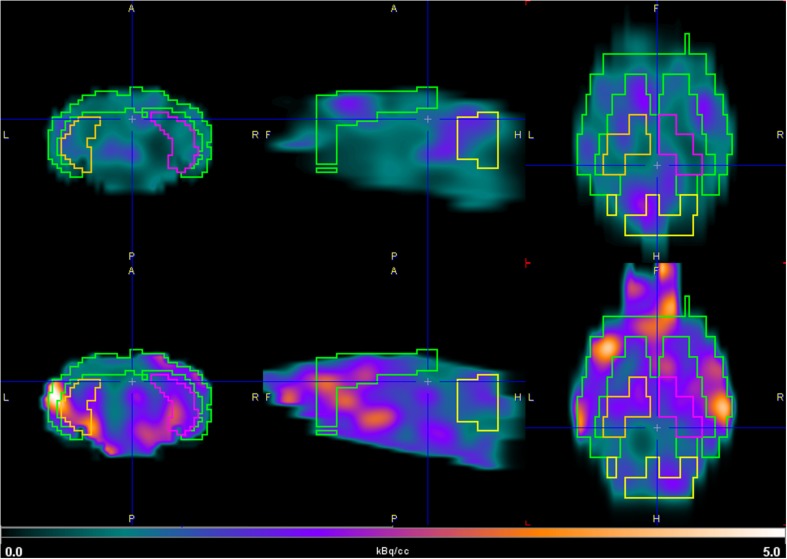
Coronal (left), sagittal (middle), and axial (right) PET images of [^18^F]2B-SRF101. Brain uptake of 3xTg mouse model (down) and control group (up) in the hippocampus and cortex between 35 and 65 min, depicted using a common scale from 0.0 to 5.0 kBq/cc.

Figure 4 shows the SUVR in different brain regions of the 3xTg mice and control group after injection of [^18^F]2B-SRF101. A significant difference in [^18^F]2B-SRF101 uptake was observed in the striatum, cortex, hippocampus, and midbrain, and this was higher in the 3xTg mice than it was in the control group (*p* < 0.05). PET images of [^18^F]2B-SRF101 are shown in Figure 5.

**FIGURE 6:**
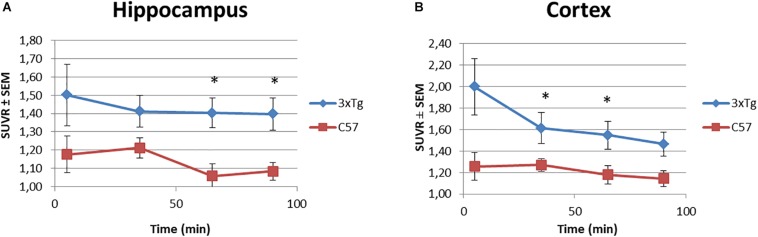
Time-SUVR curves for [^18^F]2B-SRF101 in the hippocampus **(A)** and cortex **(B)**, derived from small animal PET/CT images. Significant difference between groups are indicated by ^*^p < 0.05.

Besides, the SUVR values for hippocampus and cortex over time were analyzed. Figure 6 illustrates a tendency along the 90 min of the study, where the [^18^F]2B-SRF101 uptake in the 3xTg mice was higher than that in the control group. A statistical analysis was performed, which showed a significant difference in [^18^F]2B-SRF101 uptake from 35 to 90 min in hippocampus and from 5 to 65 min in cortex (*p* < 0.05). There was no significative difference at early frame (up to 5 min) for both tracers, which may be due to the initial blood tracer disposition.

In the comparative studies between [^18^F]2B-SRF101 and [^11^C]DED uptake in the 3xTg mice, no significant difference was observed in any of the analyzed regions (*p* > 0.05; Figure 4).

Finally, the [^11^C]DED uptake between 3xTg mice and control group was compared, and no significant difference was found in any of the analyzed regions (*p* > 0.05; Figure 4).

## Discussion

### *Ex vivo* Studies: Biodistributions

The preclinical characterisation of [^18^F]2B-SRF101 was performed after the novel compound’s ability to cross the BBB was confirmed by *ex vivo* brain fluorescence imaging (see Supplementary Material). Biodistribution profile in the control group showed a high concentration of the tracer in the liver and gut throughout the study, together with the low uptake in the kidneys and bladder (Figure 1). This suggest a primarily hepatointestinal metabolisation. These results are consistent with the following lipophilic properties of the compound, as previously determined by our group: log P_OCT_ [^18^F]2B-SRF101 = (1.88 ± 0.14) (Kreimerman et al., 2017). Figure 2 shows that the gut concentration of the tracer increased as the liver concentration decreased, which is in agreement with the previously mentioned hepatointestinal metabolisation. The high percentage of activity in gut shows that, at 30 min p.i., nearly 90% of the compound was metabolized, reflecting the absence of non-specific binding. This could also be observed in terms of the low activity concentration of the tracer in the non-target tissues, such as the heart, bones, lungs, spleen and muscles, at all analyzed times (Table 2). It is also important to note that the concentration of [^18^F]2B-SRF101 in the blood was extremely low, at 1.1 ± 0.3%ID/g at 14 min p.i.; this level decreased further throughout the study (0.16 ± 0.08%ID/g at 110 min p.i.), denoting the tracer’s quick washout in the blood. It is worth noting that there was minimal uptake in bone (∼0.05%ID/g), and this did not increase during the study. This suggests that there is no appreciable loss of [^18^F] from the compound.

The distribution of the compound in the 3xTg mouse model showed a similar pattern to that observed in the control group. In the study, a high accumulation in the gut and liver was observed, confirming the hepatointestinal metabolisation mentioned above. Non-specific binding in other tissues was not found, and a rapid washout of the tracer in the blood was also observed in this mouse model (Figure 3). Based on these results, [^18^F]2B-SRF101 showed suitable characteristics to be considered a potential PET tracer.

### *In vivo* Studies: Micro-PET/CT Imaging

Dynamic PET/CT studies with [^18^F]2B-SRF101 showed a higher uptake in some regions, particularly in cortex and hippocampus, in the 3xTg mice than in control group. These two regions are of special interest as it has been reported that this transgenic mouse model develops astrocytosis from 7 months of age, mainly in the hippocampus and cortex (Oddo et al., 2003). The mentioned difference between both groups could be observed in PET images of [^18^F]2B-SRF101 shown in Figure 5. Besides, a significant difference in [^18^F]2B-SRF101 uptake was found for hippocampus from 35 to 90 min and for cortex from 5 up to 65 min (Figure 6).

The comparative studies between [^18^F]2B-SRF101 and [^11^C]DED suggest that both tracers have different roles as astrocytosis markers in this animal model. In any case, it should be considered that both radiotracers labeled astrocytes through different molecular targets. [^18^F]2B-SRF101 was expected to detect astrocytes by a direct mechanism of binding/entry to them, so it would probably label both reactive and non-reactive astrocytes. In contrast, [^11^C]DED presents an indirect mechanism of astrocyte labeling, as it binds to MAO-B, which is mainly increased in reactive astrocytes. Therefore, astrocyte labeling with [^11^C]DED may be related to the stage of the disease, which determines the astrocyte activation level. In fact, in a recently reported study, it was shown that [^11^C]DED is a useful tool for the diagnosis of neuroinflammation in the early stages of the disease (Olsen et al., 2018). The authors stated that changes in MAO-B levels and glial fibrillary acidic protein (GFAP)/vimentin expression (astrocyte marker) do not occur simultaneously. Apparently, elevated MAO-B levels are an early event during Aβ pathology progression, but they remain practically unchanged thereafter. In contrast, GFAP and vimentin appear to increase later, most likely as a consequence of abundant Aβ plaque formation. The researchers even concluded that both markers did not co-localize to the same cell population. Thus, a single marker, such as MAO-B, GFAP or vimentin, may not be sufficient to estimate the total astrogliosis level. Based on a similar analogy with [^18^F]2B-SRF101 and [^11^C]DED, both radiotracers could provide different and complementary information at the same time. In this way, by means of a multitracer approach (Engler, 2008; Rodriguez-Vieitez and Nordberg, 2018), useful information could be obtained for the staging of the disease, thereby contributing to its early, accurate diagnosis.

Considering the postulation by some authors that SR101 labels not only astrocytes, but also mature myelinating oligodendrocytes, [^18^F]2B-SRF101’s ability to diffuse from astrocytes to oligodendrocytes should be evaluated in future works. As a breakdown of myelin is observed in AD, induced by the vulnerability of the oligodendrocytes in this disease (Cai and Xiao, 2016; Hülsmann et al., 2017), having a tracer that labels both cells (oligodendrocytes and astrocytes) could be a useful tool for detecting the chronic inflammation observed in the pathological process of AD.

Finally, we compared the [^11^C]DED uptake between 3xTg mice and control group, finding no significant difference in any of the analyzed regions (Figure 4). Recently, an evaluation of [^11^C]DED uptake on a similar AD animal model was performed (Rodriguez-Vieitez et al., 2015). In that work, the researchers demonstrated that tracer uptake decreased markedly with the age of the transgenic mice, especially in the cortex and hippocampus, while it was not age dependent in the control group. These authors even observed that [^11^C]DED activity in these areas was higher in the transgenic mice at 6 months than it was at 8 or 15 months of age. In our work, the studies were carried out with 9- and 10-month-old animals. Thus, the lower uptake observed for [^11^C]DED in comparison with [^18^F]2B-SRF101 in the 3xTg mice could be explained by the observation mentioned above. It is important to consider that several transgenic mouse models harboring familial AD mutations are currently available. They provide useful insights into the pathophysiology of the human disease, but none completely replicate it. Therefore, when analyzing the results, the model characteristics must be carefully considered for a better understanding of them.

In conclusion, the preclinical characterisation of [^18^F]2B-SRF101 was performed. Biodistribution studies allowed evaluation of the pharmacokinetic profile and verification that [^18^F]2B-SRF101 presented appropriate characteristics to become a PET diagnostic agent. A hepatointestinal metabolisation of the compound was observed, consistent with its lipophilic feature. The micro-PET/CT imaging with [^18^F]2B-SRF101 revealed that the novel tracer may be an astrocytosis marker in the animal model used in the present work.

In combination, the results suggested that [^18^F]2B-SRF101 is a promising candidate for more extensive evaluation as an astrocyte tracer. In addition, comparing the micro-PET/CT studies performed with [^18^F]2B-SRF101 and [^11^C]DED, we concluded that both tracers could provide different and complementary data. In that sense, they could be useful tools for carrying out a better assessment of the AD pathology in a multitracer approach.

## Ethics Statement

The research protocol was carried out in accordance with the National Bioethics Committee requirements and under the current ethical regulations of the national law on animal experimentation no. 18.611 (National Commission for Animal Experimentation, CNEA, Uruguay). CUDIM’s Animal Bioethics Committee approved these protocols.

## Author Contributions

HE, ES, PO, and IK designed the study. IK performed the radiolabeling and animal care. TP carried out *ex vivo* imaging studies. AR, AP, and IK performed the biodistribution and molecular imaging acquisition studies. IK, AR, and PO performed the data analysis. IK drafted the initial manuscript version. WP, AR, and PO contributed to the data analysis and manuscript revision. MI contributed with statistical analysis, data processing, and graphic design. ES and HE revised and approved the final draft of the manuscript.

## Conflict of Interest Statement

The authors declare that the research was conducted in the absence of any commercial or financial relationships that could be construed as a potential conflict of interest.

The reviewer, VK, declared a past co-authorship, with several of the authors, ES, HE, and TP, to the handling Editor.
