# Supplementary material for: Biological Assessment of a 18F-Labeled Sulforhodamine 101 in a Mouse Model of Alzheimer’s Disease as a Potential Astrocytosis Marker
Source: Front Neurosci. 2019 Jul 16;13:734. doi: 10.3389/fnins.2019.00734 (PMC6646682; doi:10.3389/fnins.2019.00734)
Supplement: Supplementary file 1 [file Table_1.DOCX]

**Biological assessment of a ^18^F-labelled Sulforhodamine 101 in a mouse model of Alzheimer’s Disease as a potential astrocytosis marker.**

Ingrid Kreimerman^1^, Ana Laura Reyes^1^, Andrea Paolino^1^, Tania Pardo^1^, Williams Porcal^1,2^, Patricia Oliver^1^, Eduardo Savio^1^, Henry Engler^1^

^1^Uruguayan Centre of Molecular Imaging (CUDIM), Radiopharmacy Department, Montevideo, Uruguay.

^2^ Department of Organic Chemistry, Faculty of Chemistry, University of the Republic (UdelaR), Montevideo, Uruguay

***Ex vivo* fluorescence imaging in mice**

1. **Methods**

Fluorescence imaging

Biodistribution studies with C57BL6J black mice (4-month old) were carried out as a screening method to determine whether 2B-SRF101 (unlabelled) could cross the BBB. To accomplish this, 2B-SRF101 was first dissolved in 0.9% NaCl (saline)/ethanol (90:10, v/v) at a concentration of 10 mg/mL. The animals were injected i.v. with SRF101 (20 mg/kg). Fifteen minutes after injection, the mice were sacrificed by cervical dislocation, and their brains were removed and coronally cut into three portions. Following sectioning, fluorescence images were acquired with a preclinical imaging system (IVIS® Lumina XR).

1. **Results**

The 2B-SRF101 solution was injected i.v., and the brain was removed 15 min later. Three coronal brain slices were analysed using a fluorescence camera. Fluorescence was observed in two of the three analysed regions (ii and iii, Figure 1).

**Figure 1**

**Discussion**

Brain imaging PET tracers are required to cross the BBB. In the current study, *ex vivo* fluorescence imaging was performed with the unlabelled 2B-SRF101 as a screening method to determine whether this new compound could cross the BBB. This enabled us to confirm that 2B-SRF101 crossed the BBB.

**Figure Legend**

**Figure 1.** Fluorescence images of 2B-SRF101 in brain slices (I: anterior; II: middle; III: posterior).
