# Supplementary figures and images for: Biological Assessment of a 18F-Labeled Sulforhodamine 101 in a Mouse Model of Alzheimer’s Disease as a Potential Astrocytosis Marker
Source: Front Neurosci. 2019 Jul 16;13:734. doi: 10.3389/fnins.2019.00734 (PMC6646682; doi:10.3389/fnins.2019.00734)

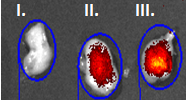

Supplement: Supplementary file 2 [file Image_1.TIF]
